# Reactive Powder Concrete Containing Basalt Fibers: Strength, Abrasion and Porosity

**DOI:** 10.3390/ma13132948

**Published:** 2020-07-01

**Authors:** Stefania Grzeszczyk, Aneta Matuszek-Chmurowska, Eva Vejmelková, Robert Černý

**Affiliations:** 1 Department of Building Materials Engineering, Technical University of Opole, 45 061 Opole, Poland; a.matuszek-chmurowska@po.edu.pl; 2 Department of Materials Engineering and Chemistry, Czech Technical University in Prague, 166 29 Prague, Czech Republic; eva.vejmelkova@fsv.cvut.cz (E.V.); cernyr@fsv.cvut.cz (R.Č.)

**Keywords:** reactive powder concrete, strength, basalt fibers, abrasion, porosity

## Abstract

The paper presents the test results of basalt fiber impact on a compressive and flexural strength, resistance to abrasion and porosity of Reactive Powder Concrete (RPC). The reasons for testing were interesting mechanical properties of basalt fibers, the significant tensile strength and flexural strength, and in particular the resistance to high temperatures, as well as a relatively small number of RPC tests performed with those fibers and different opinions regarding the impact of those fibers on concrete strength. The composition of the concrete mix was optimized to obtain the highest packing density of particles in the composite, based on the optimum particle size distribution curve acc. to Funk. Admixture of basalt fibers was used in quantity 2, 3, 6, 8 and 10 kg/m^3^, length 12 mm and diameter 18 µm. A low water-to-binder ratio, i.e., from 0.24, was obtained through application of a polycarboxylate-based superplasticizer. The introduction of up to 10 kg/m^3^ of basalt fibers to RPC mix was proved to be possible, while keeping the same w/c ratio equal to 0.24, with a slight loss of workability of the concrete mix as the content of fibers increased. It was found that the increase of the fiber content in RPC to 10 kg/m^3^, despite the w/c ratio was kept the same, caused reduction of the concrete compressive strength by 18.2%, 7.8% and 13.6%, after 2, 7, and 28 days respectively. Whereas, the flexural strength of RPC increased gradually (maximum by 15.9%), along with the fiber quantity increase up to 6 kg/m^3^, and then it reduced (maximum by 17.7%), as the fiber content in the concrete was further increased. The reduction of RPC compressive strength, along with the increase in basalt fibers content, leads to the increase of the total porosity, as well as the change in pore volume distribution. The reduction of RPC abrasion resistance was demonstrated along with the increase of basalt fibers content, which was explained by the compressive strength reduction of that concrete. A linear relation between the RPC abrasion resistance and the compressive strength involves a high determination coefficient equal to 0.97.

## 1. Introduction

Reactive Powder Concretes (RPC) are one of the most significant achievements in the field of the concrete technology in recent years [1,2]. They achieve considerable compressive strength values reaching even over 200 MPa in normal conditions, whereas with application of the hydrothermal treatment the strength values are much higher (from 300 to 500 MPa) [3,4,5]. Such high compressive strength values were achieved by elimination of coarse aggregate, which was replaced with fine ground quartz with particle size from 1 μm to 4 μm, and with sand with particle size from 200 μm to 400 μm. Apart from the increased quantity of cement (the cement content is usually 900 kg/m^3^), the use of silica fumes, finely ground quartz powder, ground granulated blast furnace slag and fly ash at a low w/b ratio (from 0.20 to 0.23) allows to provide the maximum particle packing in the concrete. Whereas a larger quantity of steel fibers, in amount from 1.5 to 3.0%, assures achievement of the relatively high flexural and tensile strength of this concrete. Steel fibers play an important role in shaping of mechanical properties of this material [6,7]. Nowadays, the interest in modification of RPC properties grows through the application of various types of fibers, including basalt ones [8], and even of polypropylene [9]. Basalt fibers, due to their mechanical properties and resistance to high temperatures, are a subject of greater interest by concrete technologists. The tensile strength of basalt fibers is 700–1680 MPa, and the Young’s modulus fluctuates within 70–90 MPa [10,11]. The advantages also include resistance to corrosion, both basic and acidic corrosion. Fibers are resistant to low and high temperatures, the range of working temperatures of application fluctuates from −260 °C to +750 °C [12].

To assess impact of the basalt fibers on the concrete properties, we have taken into account fibers content, size and the water-to-cement (w/c) ratio [13,14,15,16]. The influence of the experimental results of the basalt fibers concrete strength are ambiguous [14,17,18].

Kabay [15] tested the influence of basalt fibers in quantity 2% and 4% by mass, length 12 and 24 mm in high strength and normal strength concretes with w/c ratio equal to 0.45, and 0.60, respectively. He found that the addition of fibers caused the decrease in compressive strength and increase of the flexural strength and the fracture energy rose along with the increase in the fiber content of concrete.

Barabanshchikov and Gutskalov [13], while testing the impact of various quantity of basalt fibers (from 0.93% to 3.24% by volume) of various length 12.7 and 24.5 mm on properties of cement pastes, also found reduction of the compressive strength, whereas the increase of the flexural strength of specimens was the higher, the higher the fiber content was. It shall be noted, however, that the higher fiber content required the increase of w/c ratio from 0.31 to 0.40, which undoubtedly influenced the compressive strength. Furthermore, authors found that there was an optimum quantity of basalt fibers with the given length, which caused the highest increase of the flexural strength. In case of specimens tested, that quantity for fibers 12.7 mm long was 1.6% by volume.

Chaohua et al. [16] investigated the influence of basalt fibers, 12 and 22 mm long, in amount from 0.05% to 0.5% of the total volume of the concrete mix, with a relatively high w/c ratio equal to 0.6. They found a visible increase of flexural and tensile strength, but a slight increase of the compressive strength, and even its reduction at a later time. They stated that the content of fibers in amount of 0.3% in volume fraction was the most suitable in terms of the growth of the concrete mechanical parameters tested.

Tehmina Ayub et al. [14] observed a slight increase of the compressive strength of the High Performance Concrete with basalt fiber content 1% and 2% of volume fraction, and then the drop with a higher amount of fibers (3 vol.%). They noted the strength decrease occurred with addition of metakaolin and silica fumes. Authors observed the gradual increase of splitting tensile strength along with the increase of fiber content (from 1% to 3% by volume).

The increase of both compressive and flexural strength along with the increase of basalt fiber content from 0.25% to 1% by weight of cement, while keeping the same w/c ratio = 0.42, was found by authors of the paper [17]. The similar relation was observed by the authors of the paper [19] at the fiber content increase from 1% to 3% by weight of cement in sand basalt-fiber concrete, while keeping the w/c ratio within the range 0.32–0.35.

Not many papers on the RPC with addition of basalt fibers have been published so far. Such research was conducted by authors of the paper [8], who found that introduction of basalt fibers to RPC (w/c = 0.25) caused the increase of the compressive and flexural strength, when the content was from 1 kg/m^3^ to 3 kg/m^3^, whereas when the content of fibers increased to 5 kg/m^3^, the gradual decrease of the compressive and flexural strength occurred.

Table 1 shows results of compressive and flexural strength tests of various types of concretes, with a different content of basalt fibers from 6 mm to 25 mm long, conducted by various authors [8,13,14,15,16,17,19]. The analysis of test results of flexural strength of concretes presented in Table 1 shows that, along with the fiber content increase in the concrete, the increase in the flexural strength also occurs to a various extent. [15,16,17]. The higher growth in the flexural strength (within the range from 54% to 64%) is achieved by samples with the w/c ratio 0.34–0.42 and the higher content of fibers, above 15 kg/m^3^ [13,17]. In the case of RPC and hardened cement paste, which have a much higher content of fine particles, the flexural strength increases to a certain amount of basalt fibers in the material, and then it goes down with the further increase of the fiber content [8,13]. Application of the low w/c ratio (0.25) in RPC allows to introduce much smaller amount of fibers (up to 5 kg/m^3^), than to the cement paste with the higher w/c (0.4).

In case of the compressive strength, the increase can be also observed [17,19], as well as reduction of the strength along with the basalt fiber content in the concrete [13,14,15], and also the increase of the strength, and then its drop when a specific amount of fibers in the concrete is exceeded [8,16]. Reduction of the concrete compressive strength is observed both in case the fiber content is lower: 4.0 kg/m^3^ by 18% [15], and in case the fiber content is higher: 94 kg/m^3^ by 50% [13]. In general, the increase of fiber content makes their proper distribution in the concrete mix more difficult and may lead to higher concrete porosity, resulting in the reduction of the compressive strength [16,20]. Furthermore, the higher content of basalt fibers in the mix, to provide its workability, requires the increase of the w/c ratio, which in consequence also leads to reduction of the concrete compressive strength.

There are many factors mentioned above, which have the impact on the strength of concretes with basalt fibers, but the impact of the concrete composition cannot be ignored, including the influence of mineral admixtures to seal the cement matrix and the matrix contact with fibers.

There are multiple factors that affect the strength of concrete, and with addition of fibers, it is difficult to determine the impact on each of them individually. Based on research conducted by various authors, general trends in the change in strength can be indicated under the influence of basalt fibers introduced to the concrete. Although, most researchers use basalt fibers with an optimal length of 12 mm due to the strength of concrete [15], this parameter should also be taken into accoung when the strength of concrete with fibers are analysed.

Few papers referring to tests of RPC with basalt fibers have resulted in research to be undertaken, in order to determine the optimum quantity of basalt fibers in RPC, in terms of the compressive and flexural strength. Testing included the impact of basalt fibers on the RPC strength, resistance to abrasion and porosity.

Taking the basalt fibers resistance to high temperatures into account, the objective of tests performed was to determine composition of the RPC with the highest compressive and flexural strength, which will be subject to high temperatures, as in case of tests of RPC with steel fibers, conducted by the authors of this paper [7].

## 2. Materials and Methods

**Figure 1:**
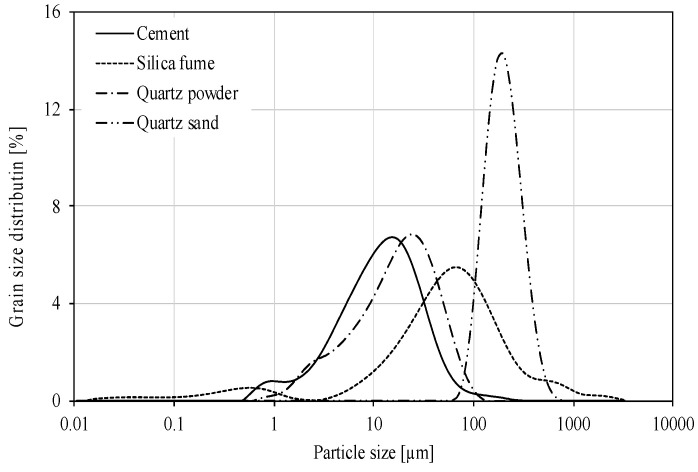
Particle size distribution of cement, silica fume, quartz powder and quartz sand.

**Figure 2:**
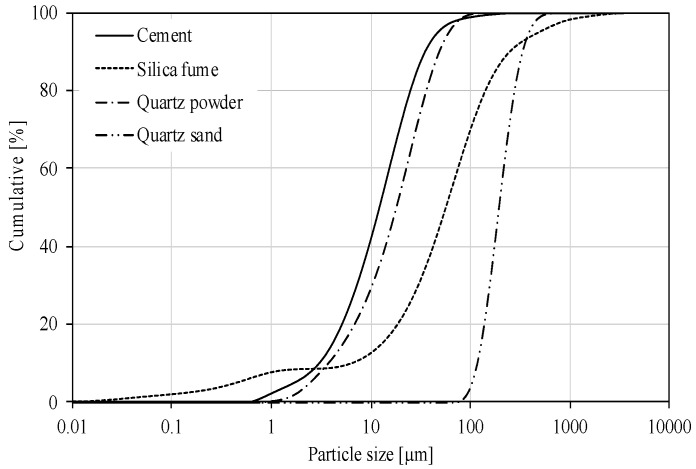
Cumulative particle size distribution of cement, silica fume, quartz powder and quartz sand.

To prepare the Reactive Powder Concrete (RPC), the following ingredients were used: Portland cement CEM I 52,5 R (Cement Plant, Rejowiec, Poland) with specific surface area 410 m^2^/kg, silica fumes, quartz powder 0/0.2 mm, quartz sand 0/0.4 mm and basalt fibers 12 mm long and diameter 18 µm. Polycarboxylate superplasticizer was added in quantity of 2.5 wt.% of cement. The chemical composition of the cement, silica fumes, quartz powder and quartz sand is given in Table 2, and Figure 1 and Figure 2 shows the particle size distribution of the cement, silica fumes, quartz powder and quartz sand determined by means of a laser size analyzer Mastersizer 3000 (Malvern, UK). Waste silica fume containing more than 62% particle > 40 μm.

**Figure 3:**
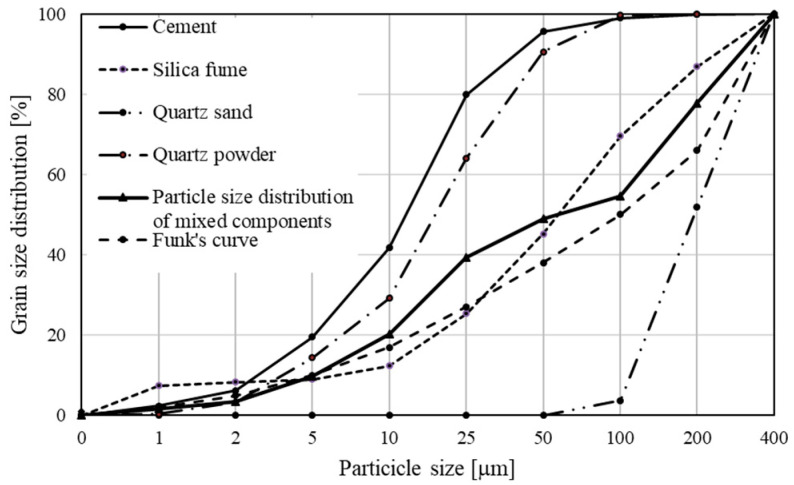
The particle size distribution of RPC mix and it’s components.

Optimization of the concrete mix composition that assured the maximum packing density of particles, was performed based on Funk and Dinger’s curve [21] (Figure 3) following formula,

(1) $$y_{i}=\left(\frac{d_{i}^{n}-d_{\mathrm{Min}}^{n}}{d_{\mathrm{Max}}^{n}-d_{\mathrm{Min}}^{n}}\right)\times 100\%,$$

where y_i_—cumulative percentage of i-fraction content, d_i_—i-fraction diameter [μm], d_Max_—maximum particle diameter [μm], d_Min_—minimum particle diameter [μm], n—constant value equal to 0.37.

For that purpose, also other computational models are used [22,23].

Composition of RPC mixes is given in Table 3. The content of basalt fibers in the mix were 2; 3; 6; 8 and 10 kg/m^3^, at the same w/c ratio amounting to 0.24.

The particle size distribution of the concrete was determined by means of the particle size laser analyzer Mastersizer 3000 within the range 0.01–3500 μm.

X-ray diffraction (XRD) tests were conducted by means of Philips X’PertSystem diffractometer (Amsterdam, The Netherlands). The measurement was conducted within a range from 5° to 60° 2θ. CuKα radiation was used.

Tests of RPC consistence were performed in line with PN-EN 1015-3 standard (Methods of test for mortar for masonry. Part 3: Determination of consistence of fresh mortar (by flow table)) [24]. Consistence was determined based on measurement of the concrete flow diameter.

The tests of compressive strength and flexural strength of RPC specimens were performed in line with PN-EN 1015-11 standard (Methods of test for mortar for masonry. Part 11: Determination of flexural and compressive strength of hardened mortar) [25]. Tests were performed on specimens 40 mm × 40 mm × 160 mm each time. The flexural strength was calculated according to the following formula,

(2) $$f=1.5\frac{\mathrm{Fl}}{{\mathrm{bd}}^{2}},$$

where f—flexural strength [MPa], F—maximum load [N], l—distance between axes of cylinders [mm], b—width of specimen [mm], d—height of specimen [mm].

The compressive strength was calculated according to the following formula:

(3) $$f_{c}=\frac{F}{A_{c}},$$

where: fc—compressive strength [MPa], F—maximum load [N] and A_c_—section area of specimen [mm^2^].

Resistance to abrasion of RPC specimens was tested in accordance with PN-EN 1338 standard (Concrete paving blocks. Requirements and test methods) [26]. Measurement of abrasion was performed on Böhme’s disc (EMEL, Poland). Specimens were subject to abrasion load equal to 294 ± 3 N during 16 cycles. The abrasion resistance was calculated according to the following formula,

(4) $$A=∆V=\frac{∆m}{\rho_{R}}∆l\times 5,$$

where ∆m—specimen mass loss (wastage) after 16 cycles [g], ρ_R_—specimen density [g/mm^3^].

Infrared Spectra (IR) of basalt fiber specimen in the form of potassium bromide pellets (5 mg/500 mg KBr) recorded on Thermo Nicolet spectrophotometer (ThermoFisher Scientific, Waltham, MA, USA), Nexus model.

The microstructure of RPC was tested by scanning electron microscopy (SEM). Analyses were performed by means of the scanning microscope NOVA NANO SEM 200 (FEI Europe B.V., Eindhoven, The Netherlands). Observations were conducted with magnification from 200 to 10,000. Specimens for testing were prepared by spraying a layer of gold on the surface in high vacuum conditions. Energy Dispersive X-Ray Spectra (EDS) were obtained for selected points.

Testing of RPC porosity were performed with the use of a mercury porosimeter PoreMaster 60 (Quantachrome Instruments, Boynton Beach, FL, USA), within a pressure range from 1 to 400 MPa. The results of the tests were presented in a form of differential curves and volumetric distribution curves of pores with different size.

## 3. Results and Discussion

### 3.1. Test of Basalt Fibers

Identification tests of basalt fibers were conducted by means of XRD test, infrared spectroscopy and scanning electron microscopy.

**Figure 4:**
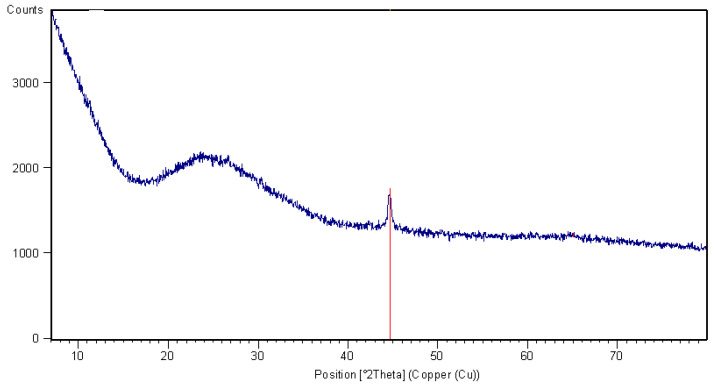
XRD pattern of basalt fibers.

An XRD pattern of basalt fibers specimen is presented in Figure 4. A reflex of metallic iron occurred in XRD pattern next to a significant background rise associated with amorphous glass. It is very likely that, during the melting of basalt dust, iron crystallized out without binding with the glass. Notably, basalt contains quite a lot of magnetite, its reduction and crystallization in a form of metallic iron cannot be excluded.

**Figure 5:**
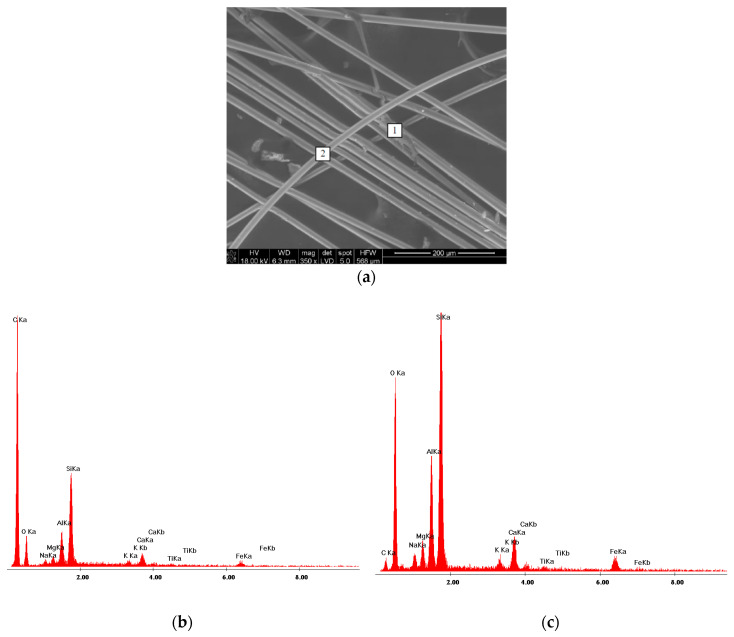
(**a**) SEM of basalt fibers; (**b**) EDS analysis in point 1; (**c**) EDS analysis in point 2.

Figure 5a shows SEM image of basalt fibers. As you can see, roughness occurs here and there on the smooth surface of fibers. EDS spectra (Figure 5b,c) made in two different points on basalt fibers (points 1 and 2)—Figure 5a) demonstrated the presence of carbon. A much lower intensity of a line belonging to the carbon is observed on the EDS spectrum of the smooth surface of the fiber (Figure 5c) than on the rough surface (Figure 5b). The presence of carbon on basalt fibers surface may indicate that fibers are coated with the organic substance, more quantity of it in some places.

**Figure 6:**
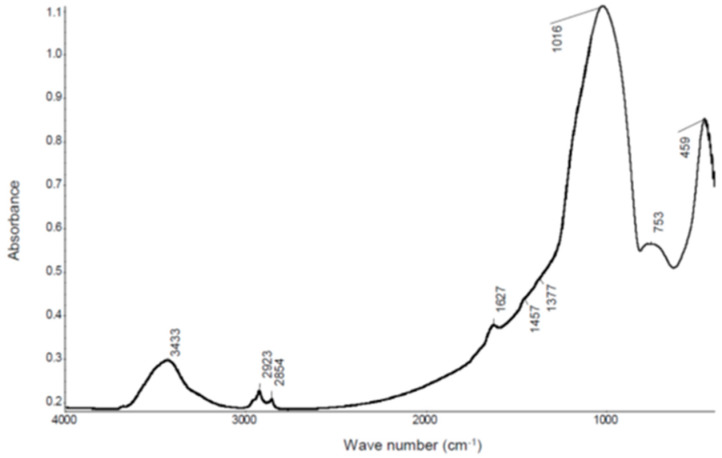
IR spectra of basalt fibers in range from 4000 to 500 cm^−1^.

**Figure 7:**
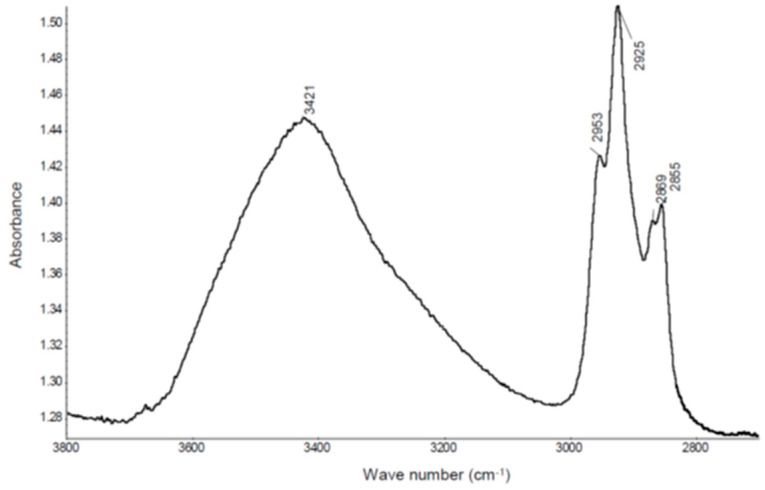
IR spectra of basalt fibers in range from 3800 to 2700 cm^−1^.

IR spectroscopy tests were performed to identify the organic substance. The IR spectra of basalt fibers is presented in Figure 6, whereas Figure 7 shows a fragment of the spectra characteristic for vibration of aliphatic groups of CH_2_ and CH_3_.

Analysis of IR spectra demonstrated the presence of bands belonging to CH_2_ and CH_3_ groups in the spectrum: 2953 cm^−1^—_STR/AS_CH_3_, 2925 cm^−1^—_STR/AS_CH_2_, 2869 cm^−1^—_STR/S_CH_3_, 2855 cm^−1^—_STR/S_CH_2_. These bands are well visible in Figure 7. Furthermore, in the IR spectrum (Figure 6) there are also trace bands coming from deformation (bending) vibration of CH_3_, CH_2_ groups (1457 cm^−1^) and scissor vibration of CH_3_ groups (1377 cm^−1^). The results of the above testing indicate that the surface of basalt fibers contains a hydrocarbon modifier. It is also supported by results of the SEM testing. EDS spectra made in two different points: 1 (Figure 5b) and 2 (Figure 5c) marked in the microscope image of basalt fibers (Figure 5a) showed that the line belonging to carbon is much more intense in point 1 than on the fiber smooth surface (point 2). Point 2 clearly shows presence of the modifier on the fiber surface.

The lack of bands coming from oxygen functional groups in the spectra: $\begin{array}{c} \|\\ -C- \end{array}$, $\begin{array}{c} -C-O-C- \end{array}$, $-\begin{array}{c} \|\\ C \end{array}-O-$, which may come from the substance that modifies the fibers surface, allows to suspect that the hydrocarbon (oil) is the modifier itself or siloxane ethers (silicones) e.g., silicone oil.

The IR spectra shows an intensive, broad band belonging to siloxane groups Si–O–Si _STR/AS_ (1016 cm^−1^). Unfortunately, based on the presence of this band, it cannot be stated definitively whether the silicone oil is the modifier, as basalt fibers also contain Si–O–Si groups. The authors of the paper [10] write about modification of fiber surface with silicon oil, in order to increase the adhesion to the polymer matrix. The presence of oil on the surface of fibers, found in testing (IR and SEM), undoubtedly facilitates dispersion of fibers in the RPC mix, but it may have an adverse effect on adhesion of basalt fibers to the cement matrix, which may contribute to reduction of the concrete compressive strength.

### 3.2. Consistence of Concrete Mix

**Figure 8:**
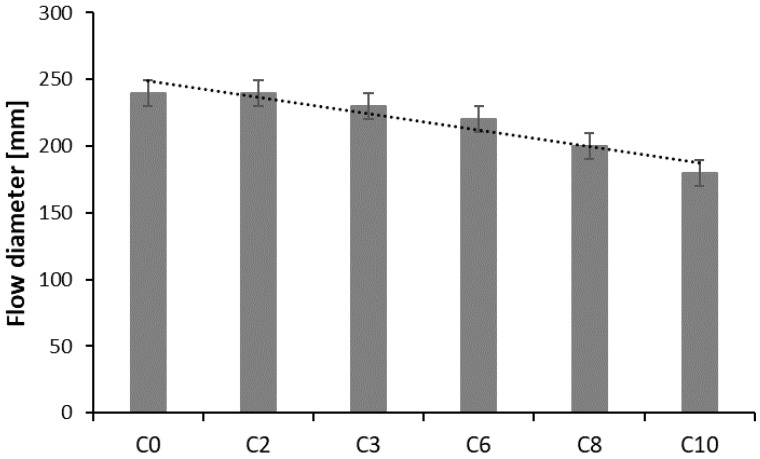
Impact of basalt fibers quantity on RPC flow diameter.

Results of RPC mix consistence tests after 15 min with a flow table test method [24], are presented in Table 4 and Figure 8.

As data in Table 4 show, the increase of the basalt fibers content from 2 kg/m^3^ to 10 kg/m^3^ in RPC mix, while keeping the same w/c = 0.24, causes reduction of the flow diameter from 240 mm to 180 mm, which indicates a drop in concrete mix flowability. It results from a commonly known fact of a larger water demand of concrete mixes with the addition of fibers, in order to obtain the required workability.

### 3.3. Compressive and Flexural Strength

**Figure 9:**
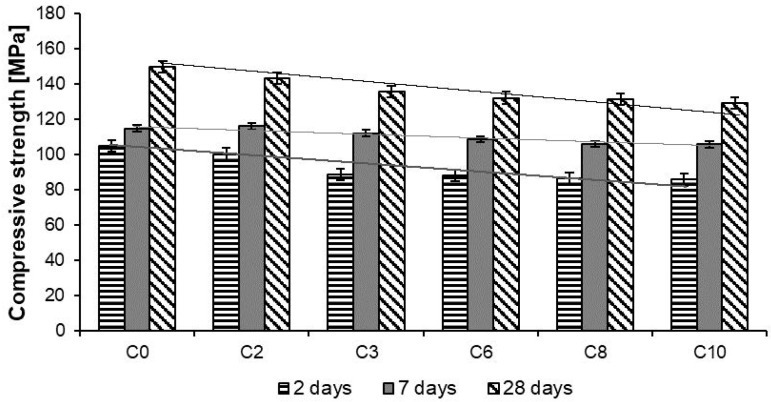
Compressive strength of RPC.

**Figure 10:**
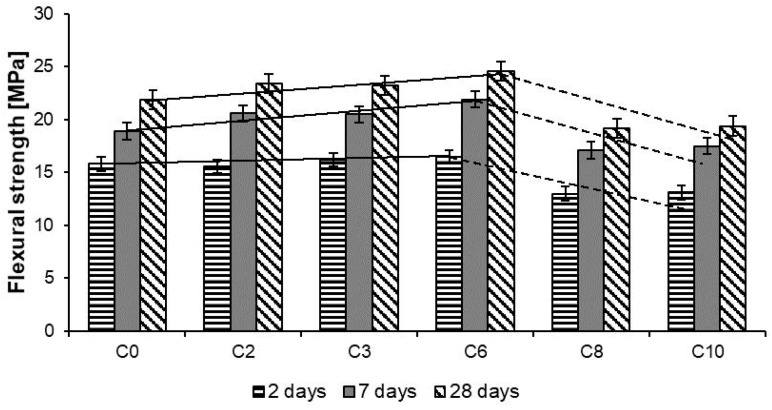
Flexural strength of RPC.

The results of compressive strength tests of Reactive Powder Concretes are shown in Table 5 and Figure 9, and results of flexural strength tests in Table 6 and Figure 10.

Based on data given in Table 5 and in Figure 9 it appears that addition of basalt fibers, in quantity from 2 kg/m^3^ to 10 kg/m^3^, while keeping the same w/c ratio, causes a reduction in the compressive strength, which is the higher in value, the higher the fiber content is. The lowest compressive strength values were obtained by concrete with the highest content of fibers (10 kg/m^3^). The elimination of w/c coefficient influence the compressive strength of RPCs with different amounts of basalt fibers, which indicates that the main factor determining the strength is the amount of fibers. Increasing the amount of fibers in the concrete mix makes their dispersion difficult, which in consequence, does not allow a homogeneous microstructure of the material to be obtained. This can cause an increase in porosity, resulting in a decrease in the compressive strength of the concrete.

The analysis of RPC flexural strength test results, while keeping the same w/c ratio, showed that as the content of basalt fibers increased from 2 kg/m^3^ to 6 kg/m^3^, increases the RPC flexural strength. Whereas, when the quantity of fibers was higher (8 kg/m^3^ and 10 kg/m^3^), a reduction of the concrete flexural strength was observed, but the difference in RPC strength with 8 kg/m^3^ and 10 kg/m^3^ content is small (Table 6). To sum up, it can be stated that there is a certain amount of basalt fibers added to RPC, at which it reaches the highest flexural strength. For the RPC tested this value is 6 kg/m^3^. A similar phenomenon was observed by authors of the paper [8], except that the quantity of basalt fibers at which they found the highest flexural strength was 3 kg/m^3^. To explain that phenomenon, it would be advisable to perform testing of basalt fibers distribution in the RPC. However, proposing a model of basalt fiber distribution in the RPC composite space, based on statistical grounds, analogically to steel fibers, is hindered. The methods applied for testing of steel fibers distribution in concretes, e.g., electromagnetic induction and ultrasonic wave propagation [27], will not meet their task in case of basalt fibers.

### 3.4. Porosity

A few factors have the impact on RPC compressive strength reduction along with the increase of the fiber content [15,18]. One of them is the concrete porosity. According to [16], admixion of the basalt fibers increases the concrete porosity. In case of RPC tested it was found that introduction of basalt fibers caused the increase of the total porosity along with the growth of fibers content, as well as a change of pores volume distribution (Table 7). A reduction in the total porosity in time was also observed.

**Figure 11:**
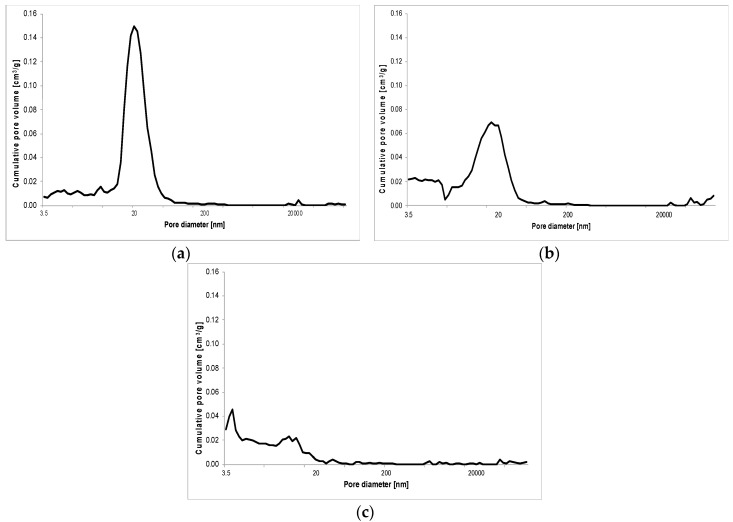
Pore volume distribution vs. pore diameter—RPC without basalt fibers (C0) after 2 (**a**), 7 (**b**) and 28 (**c**) days.

**Figure 12:**
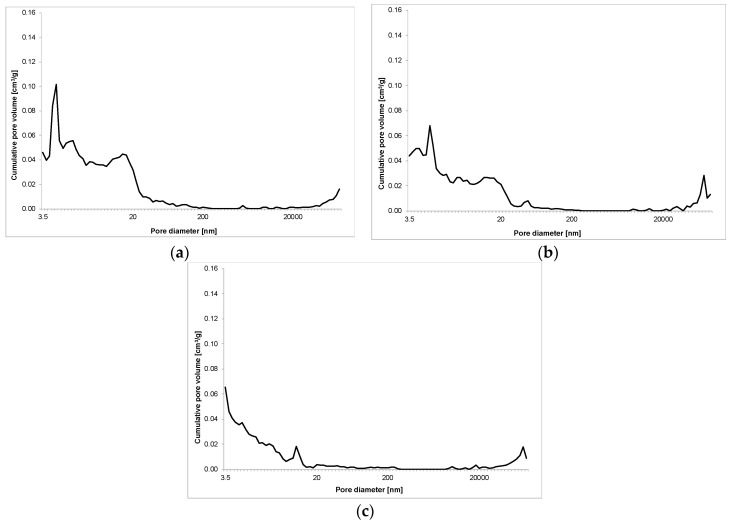
Pore volume distribution vs. pore diameter—RPC with basalt fibers (C2) after 2 (**a**), 7 (**b**) and 28 (**c**) days.

**Figure 13:**
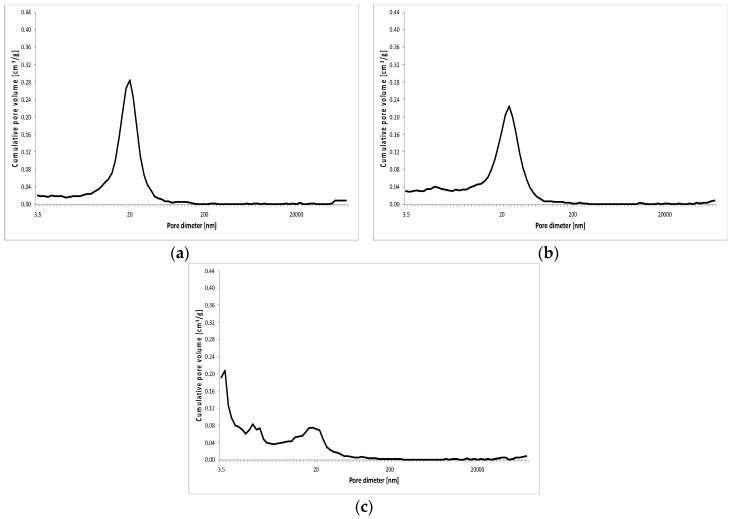
Pore volume distribution versus pore diameter—RPC without basalt fibers (C6) after 2 (**a**), 7 (**b**) and 28 (**c**) days.

**Figure 14:**
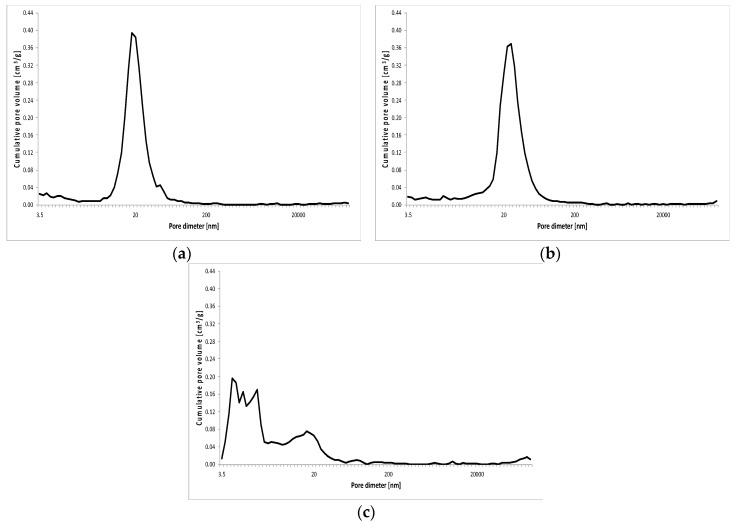
Pore volume distribution vs. pore diameter—RPC with basalt fibers (C10) after 2 (**a**), 7 (**b**) and 28 (**c**) days.

The results of porosity testing of RPC with and without basalt fibers in quantity of: 2 kg/m^3^, 6 kg/m^3^ and 10 kg/m^3^ are presented in Figure 11, Figure 12, Figure 13 and Figure 14 and Table 7.

As shown in Figure 11, Figure 12, Table 7, the total porosity of RPC without basalt fibers and a small amount of fibers (2 kg/m^3^) is comparable after 2, 7 and 28 days of curing of specimens. Whereas, the distribution of pore volume is different. RPC with basalt fibers contains definitely more pores with a larger diameter above 20,000 nm, but fewer smaller pores with the diameter from 200 nm to 2000 nm compared to the concrete without fibers, while keeping almost the same total porosity in both concretes. After 2 days of curing, the concrete with fibers showed nearly five times higher amount of pores with diameter above 20,000 nm and about five times lower amount of pores within the range 20–200 nm, compared to the content of those pores in the concrete without fibers. In the later period (7 and 28 days) those differences were smaller. In addition, a reduction in total porosity in time (from 2 to 28 days) can be clearly seen.

The addition of basalt fibers to RPC in quantity of 6 kg/m^3^ and 10 kg/m^3^, causes a definite growth of RPC total porosity, even by over 10 vol.% along with the increase of the fibers content, but similar to the lower fiber content, the total porosity is reduced over time. A reduction in pore content < 20 nm can be observed, as well as a definite higher amount (by several times) of pores within a range 20–200 nm, compared to the content of these pores in the specimen with 2 kg/m^3^ of basalt fibers. After 7 days, the quantity of these pores is four or even five times higher for RPC specimens with fiber content 6 kg/m^3^ and 10 kg/m^3^, respectively. Whereas after 28 days, a visible growth of pores < 20 nm is observed with simultaneous reduction of pores within 20–200 nm. The content of pores > 20,000 nm is generally lower than 10 vol.% for RPC with basalt fiber content 6 kg/m^3^ and 10 kg/m^3^ in the time tested, after 2, 7 and 28 days.

In general, the content of pores within the ranges 200–2000 nm and 2000–20,000 nm, both in RPC without fibers, as well as with the various content of basalt fibers, is relatively small. In the first case it generally does not exceed 3.0 vol.% and in the second one 2.0 vol.%.

The change in pore volume distribution in RPC, along with an increase in the basalt fibers content, most probably results from the impediment of fiber dispersion in the concrete mix with a very low w/c ratio, and may have the impact on the concrete strength. Cement pastes compressive strength reduction is related to the growth of pore volume with diameters larger than 20 nm [28,29]. The reduced content of pores < 20 nm with simultaneous increase of pores in the range 20–200 nm, with the basalt fibers content above 2 kg/m^3^ in RPC, as found in this paper, may contribute to the reduced compressive strength of RPC with the higher fibers content.

Furthermore, the growth of RPC total porosity found along with the increase of basalt fibers content, while keeping the constant water-cement ratio, is one of significant factors having an impact on the reduction of the concrete compressive strength. An increase in porosity, along with the increase in basalt fibers in the concrete, as well as the effect of that phenomenon on its strength, was observed by the authors of the paper [16]. Whereas, authors of the paper [15,16] emphasize that introduction of the larger quantity of basalt fibers to the concrete mix, generally requires the increase of the w/c ratio to provide concrete workability, and in consequence it affects the reduction of the concrete compressive strength.

### 3.5. Abrasion

**Figure 15:**
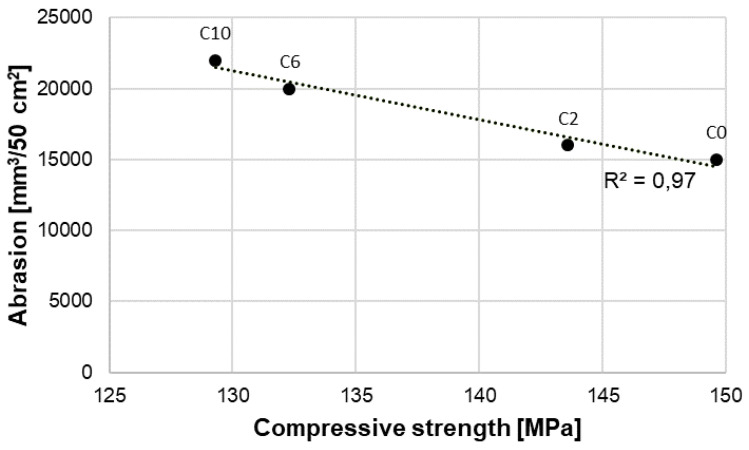
Abrasion—compressive strength relation.

**Figure 16:**
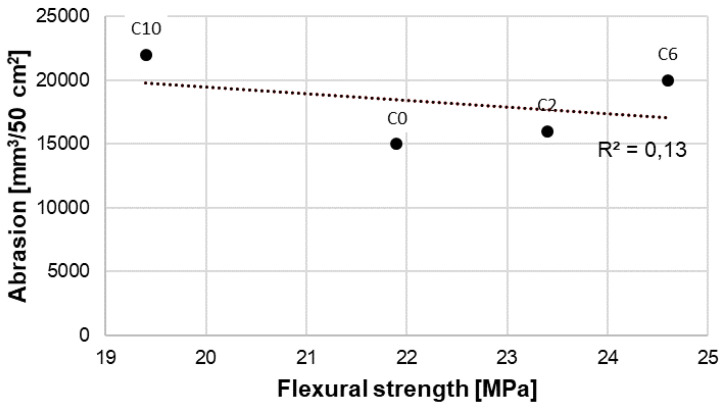
Abrasion—flexural strength relation.

Results of abrasion tests of RPC without fibers and containing 2, 6 and 10 kg/m^3^ of basalt fibers are presented in Table 8 and Figure 15 and Figure 16.

The analysis of abrasion test results of RPC with addition of basalt fibers showed that the resistance to abrasion was associated with the concrete compressive strength (Figure 15). Increased compressive strength of a concrete causes enhanced resistance to abrasion. The highest resistance to abrasion was reached by RPC with basalt fiber content of 2 kg/m^3^, which showed the highest compressive strength. Whereas, as the content of basalt fibers in RPC grows, the resistance to abrasion is reduced, which is associated with reduction of the compressive strength of this concrete. No linear relation was found between the abrasion resistance of RPC with basalt fibers and the flexural strength (Figure 16). The above confirms the value of R^2^ determination coefficients, which is high (R^2^ = 0.97) for abrasion dependence on the compressive strength (Figure 15), and low (R^2^ = 0.13) for abrasion dependence on the flexural strength (Figure 16). It means that no linear relationship between the flexural strength and abrasion occurs, which was found also by means of Pearson correlation coefficient equal to −0.32 (weak correlation). Whereas, for data presented in Figure 15, Pearson correlation coefficient is equal to −0.96.

Different results of testing were obtained by authors of the paper [15], who found that abrasion of the RPC containing basalt fibers depends on the flexural strength rather than on the compressive strength.

**Figure 17:**
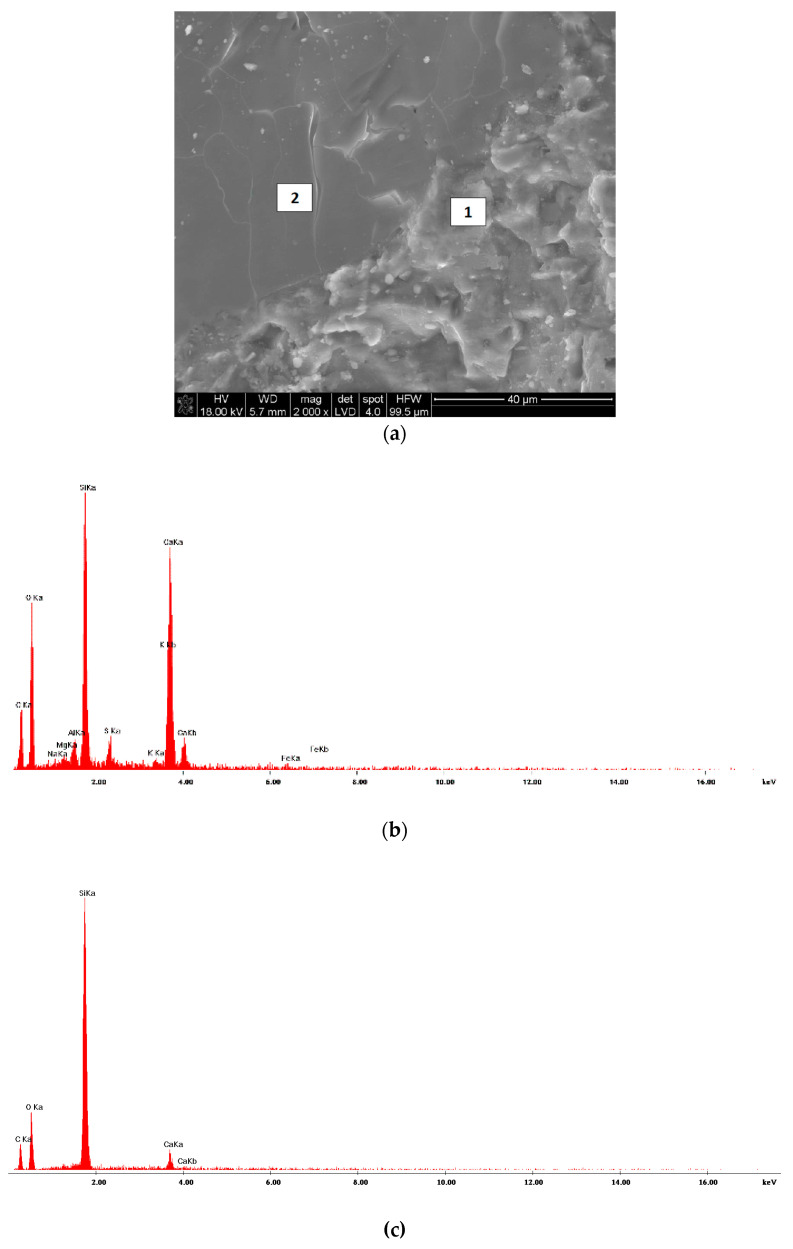
(**a**) RPC microstructure. Very good adhesion of C-S-H (1) on the quartz grain (2) is visible; (**b**) EDS analysis in point 1; (**c**) EDS analysis in point 2.

**Figure 18:**
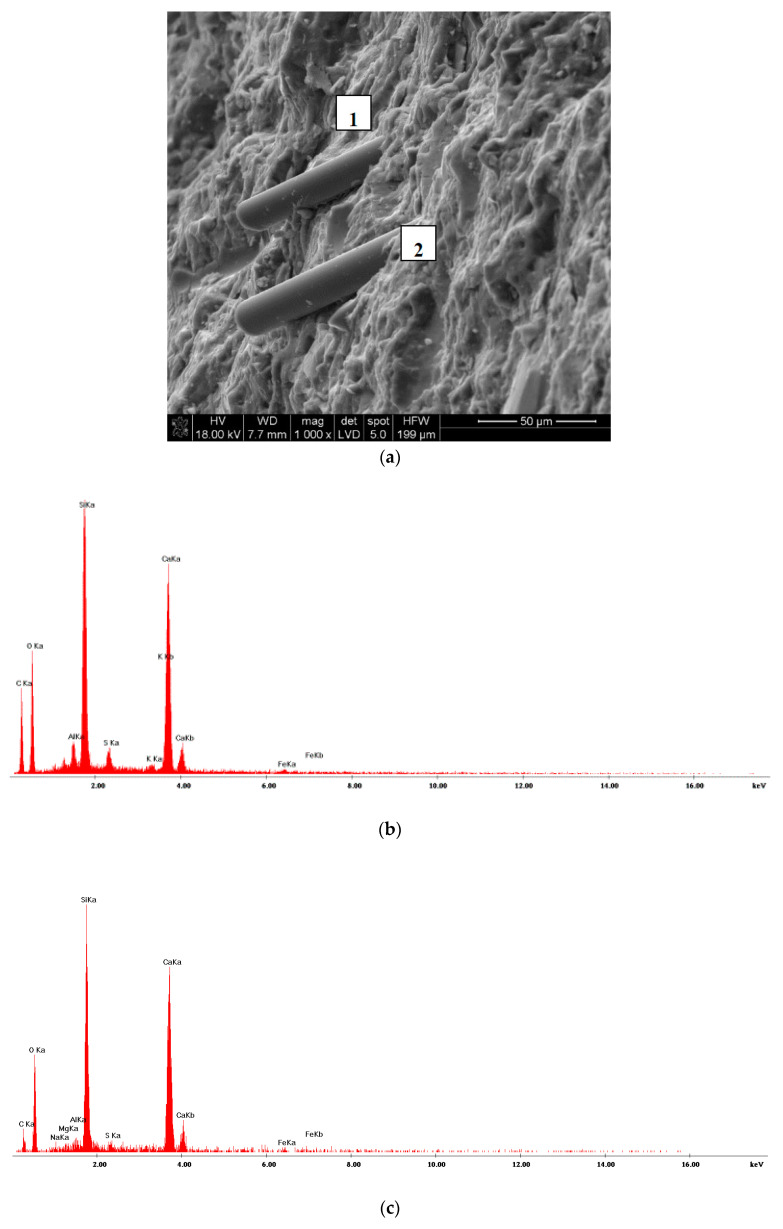
(**a**) RPC microstructure. Visible basalt fiber and C-S-H phase well adhering to the fiber (1, 2); (**b**) EDS analysis in point 1; (**c**) EDS analysis in point 2.

**Figure 19:**
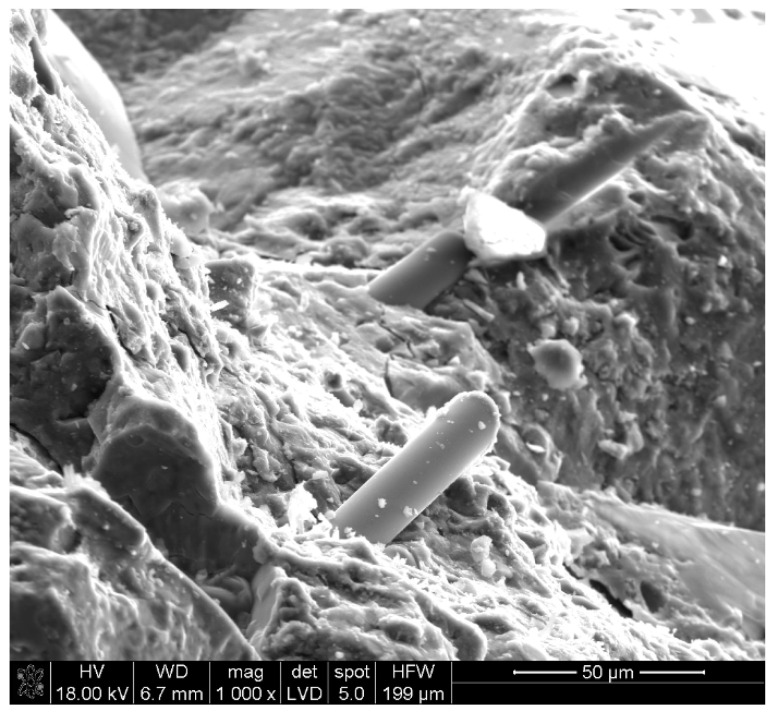
RPC microstructure. Visible parallel arrangement of basalt fibers.

The microstructure of RPC with basalt fibers in amount of 2 kg/m^3^ showed the highest compressive strength after 28 days of curing is presented in Figure 17, Figure 18 and Figure 19.

The observation of RPC microstructure under the scanning microscope showed the presence a high content of C-S-H phase bonded to quartz grains (Figure 17) and basalt fibers, which in general, are arranged parallel to each other in the cement matrix (Figure 18 and Figure 19). The high content of C-S-H, analogous to RPC with steel fibers [30], is mainly caused by a large quantity of cement and addition of silica fumes and quartz powder, known for their high reactivity to calcium ions.

## 4. Conclusions

The paper impact of basalt fiber on the properties of the RPC mix and hardened concrete.

The results obtained show that the addition of basalt fibers to the RPC leads to a decrease in the workability of concrete mix. It results from a commonly known fact of a larger water demand of concrete mixes with addition of fibers in order to get the required workability.

While keeping the same w/c ratio equal to 0.24, the compressive strength of RPC containing basalt fibers reduces along with the increase of the fiber content in the concrete. The highest drop of the compressive strength from 15.5% to 18.2% with the fiber content increase from 2 kg/m^3^ to 10 kg/m^3^ is observed after two days. A similar relation is observed after 7 and 28 days of specimen curing, however, the reduction of the compressive strength along with the fiber content increase occurs in a lesser extent over time and it is: 7.8%, and 13.6%, respectively for specimens containing the highest quantity of basalt fibers (10 kg/m^3^). A reduction of RPC compressive strength is caused by the presence of a larger quantity of pores along with the increase of the fiber content in this concrete. Whereas, the clear reduction of the total porosity of specimens over time (from 2 to 28 days), due to the increase of cement hydration products formed, weakens the impact of fiber quantity on reduction of RPC compressive strength.

Introduction of basalt fibers to RPC in quantity up to 6 kg/m^3^ causes a slight gradual growth in the flexural strength (maximum by 15.9%). Whereas, with the higher content of fibers (8 and 10 kg/m^3^), reduction of the flexural strength occurs (maximum by 17.7%), but the differences in flexural strength of RPC specimen with the content 8 kg/m^3^ and 10 kg/m^3^ are minimal and they are from 0.1 MPa to 0.4 MPa. The increase of the flexural strength along with the increase of the fiber content up to 6 kg/m^3^ goes up over time and after 2, 7 and 28 days it amounts to: 4.4%, 15.9%, and 12.3%, respectively. The higher content of basalt fibers in the RPC mix at the lower w/c ratio used (0.24), deteriorates their dispersion, which in consequence, may cause a reduction of the flexural strength.

The reduced resistance to abrasion resistance of RPC along with the increase of basalt fibers content is caused by the reduction of the concrete compressive strength. A strong relationship was found between the RPC resistance to abrasion and its compressive strength. The determination coefficient R^2^ for this relationship is 0.97. Whereas, there was no significant relationship between the resistance to abrasion and the flexural strength (R^2^ = 0.13). The compressive strength is generally recognized as the most important factor that affects the concrete resistance to abrasion. The results abrasion resistance tests of RPC with basalt fibers also confirmed this relationship.

The introduction of basalt fibers to RPC causes the increase of the total porosity, as well as the change in distribution of pores as the content of basalt fibers grows. At the fiber content of 2 kg/m^3^, the content of larger pores > 20,000 nm grows, and the content of 20–200 nm pores goes down. Whereas at the higher content of fibers (6 kg/m^3^ and 10 kg/m^3^), reduction of pores below 20 nm occurs, as well as the increase of pores within a range 200–2000 nm, but the content of pores above 20,000 nm goes down. The above changes in the total quantity of pores and their distribution correspond well with the reduction of RPC compressive strength, as the content of basalt fibers in that concrete increases.

## Figures and Tables

**Table 1:** Impact of basalt fibers quantity on compressive and flexural strength according to various authors.

Authors	Type of Concrete	w/c	Length of Basalt Fibers (mm)	Fiber Content		Compressive Strength *f_c_* (MPa) After 28 Days	Flexural	Comments
							Strength *f_cf_* (MPa)	
				By Volume (%)	By Weight (kg/m^3^)		After 28 Days	
Chonghai D., Xinwei M. [8]	RPC	0.25	12		0	86	17.3	*f_c_*–increase from 0 to 3 kg/m^3^, above decrease
					2	101	19.8	*f_cf_*–increase from 0 to 3 kg/m^3^, above decrease
					3	115	20.4	
					4	106	19	
					5	102	20	
Barabanshchikov Y., Gutskalov I. [13]	Cement Paste	0.31	12.7	0	0	72.4	6.3	*f_c_*–decrease from 0 to 94 kg/m^3^
		0.34		0.93	26.9	47.8	10.9	*f_cf_*–increase from 0 to 66.4 kg/m^3^, above decrease
		0.36		1.61	47.6	41.5	15.9	
		0.38		2.29	66.4	37.2	16.8	
		0.4		3.24	94	36.2	14.9	
Ayub T. et al. [14]	HPC	0.4	25	0	0	71.9	-	*f_c_*–increase from 0 to 60 kg/m^3^, above decrease
				1	30 *	73.5	-	
				2	60 *	74.1	-	
				3	90 *	65.1	-	
Kabay N. [15]	HSC	0.45	12	0	0	69.3	7.7	*f_c_*–decrease from 0 to 4 kg/m^3^
				0.07	2	62.4	8	*f_cf_*–increase from 0 to 4 kg/m^3^
				0.14	4	56.9	8.5	
		0.6		0	0	49.4	6.3	
				0.07	2	44.8	6.7	
				0.14	4	46.7	7.1	
Jiang C. et al. [16]	FRC	0.6	12	0	0	45.1	7.6	*f_c_*–increase from 0 to 3 kg/m^3^, above decrease
				0.05	1.5 *	46.7	8.1	*f_cf_*–increase from 0 to 3 kg/m^3^, above decrease
				0.1	3.0 *	47.2	8.2	
				0.3	9.0 *	46.3	8.4	
				0.5	15.0 *	45	8.3	
Ahmed T. et al. [17]	FRC	0.42	6–10	0	0	34.6	3.7	*f_c_*–increase from 0 to 30 kg/m^3^
				0.25	7.5 *	37.5	5.8	*f_cf_*–increase from 0 to 30 kg/m^3^
				0.5	15.0 *	42.7	7.4	
				0.75	22.5 *	50.4	9.5	
				1	30.0 *	64.1	10.2	
Morozov N.M. et al. [19]	Sand FRC	0.32		0%	0	92	-	*f_c_*–increase from 0 to 16.5 kg/m^3^
				2% **	11	113	-	
				3% **	16.5	121	-	

* A conversion factor of amount basalt fibers was used: 0.1% by volume = 3 kg/m^3^ according to [13]. ** By weight of cement.

**Table 2:** Chemical composition of RPC ingredients (% by mass).

Ingredient	SiO_2_	Fe_2_O_3_	Al_2_O_3_	CaO	MgO	SO_3_	Na_2_O	K_2_O
Cement	21.83	2.00	4.38	65.68	0.93	3.29	0.29	0.32
Silica fume	93.3	2.3	1.5	1.0	-	-	-	1.6
Quartz powder	99.0	0.05	0.29	< 0.1	< 0.1	-	0.2	< 0.1
Quartz sand	98.6	0.03	0.75	-	-	-	-	-

**Table 3:** Mix proportion of RPC [kg/m^3^].

Cement	Silica Fume	Quartz Powder	Quartz Sand	Superplasticizer	Water	w/c
876	175	105	902	22	210	0.24

**Table 4:** Flow diameter of concrete mix [mm].

RPC	C0	C2	C3	C6	C8	C10
Fibers content [kg/m^3^]	0	2	3	6	8	10
w/c	0.24	0.24	0.24	0.24	0.24	0.24
Flow diameter	240	240	230	220	200	180

**Table 5:** Compressive strength of RPC containing basalt fibers [MPa].

RPC		C0	C2	C3	C6	C8	C10
Fiber content [kg/m^3^]		0	2	3	6	8	10
w/c		0.24	0.24	0.24	0.24	0.24	0.24
Compressive strength after days	2	105.0	100.4	88.7	88.3	86.7	85.9
	7	114.9	116.2	112.2	108.9	106.2	105.9
	28	149.6	143.6	135.6	132.3	131.5	129.3

**Table 6:** Flexural strength of RPC containing basalt fibers [MPa].

RPC		C0	C2	C3	C6	C8	C10
Fiber content [kg/m^3^]		0	2	3	6	8	10
w/c		0.24	0.24	0.24	0.24	0.24	0.24
Flexural strength after days	2	15.8	15.6	16.2	16.5	13.0	13.1
	7	18.9	20.6	20.5	21.9	17.1	17.5
	28	21.9	23.4	23.2	24.6	19.2	19.4

**Table 7:** Total porosity and volumetric percentage of pores of different sizes in RPC.

Type of Concrete	Total Porosity [%] After Days		Percentage of Pores [%]				
			< 20 nm	20–200 nm	200–2000 nm	2000–20,000 nm	<20,000 nm
C0	2	10.9	39.8	55.5	2.5	0.5	2.0
	7	8.2	64.9	25.1	1.5	0.2	8.2
	28	4.4	77.1	8.0	3.6	2.5	8.2
C2	2	10.6	73.9	12.6	0.8	0.6	10.8
	7	8.5	64.9	12.2	1.5	0.4	20.4
	28	4.0	62.5	9.1	14.6	1.8	11.9
C6	2	21.8	52.1	38.7	1.4	0.8	6.7
	7	10.4	39.2	52.5	2.3	0.8	4.8
	28	15.7	69.4	16.6	2.4	0.8	10.5
C10	2	21.2	46.5	45.3	2.4	0.6	5.4
	7	25.8	22.9	68.6	3.4	1.0	3.8
	28	15.5	74.1	11.9	2.7	1.7	8.0

**Table 8:** Abrasion and strength of RPC with basalt fibers.

RPC	C0	C2	C6	C10
Fiber content [kg/m^3^]	0	2	6	10
Abrasion [mm^3^/50 cm^2^]	15,000	16,000	20,000	22,000
Compressive strength after 28 days [MPa]	149.6	143.6	132.3	129.3
Flexural strength after 28 days [MPa]	21.9	23.4	24.6	19.4
